# The psychological impact of instrumental activities of daily living on people with simulated age-related macular degeneration

**DOI:** 10.1192/bjo.2022.558

**Published:** 2022-08-08

**Authors:** Anne Macnamara, Scott Coussens, Celia Chen, Victor R. Schinazi, Tobias Loetscher

**Affiliations:** Cognitive Ageing & Impairment Neurosciences Laboratory, UniSA Justice & Society, University of South Australia, Australia; Department of Ophthalmology, Flinders Medical Centre, Flinders University, Australia; Department of Psychology, Faculty of Society & Design, Bond University, Australia; and Future Health Technologies, Singapore-ETH Centre, Campus for Research Excellence and Technological Enterprise (CREATE), Singapore

**Keywords:** Age-related macular degeneration, visual impairment, activities of daily living, stress, anxiety

## Abstract

**Background:**

People with age-related macular degeneration (AMD) can report reduced mental health. There is also evidence that they struggle with daily tasks because of vision loss.

**Aims:**

The purpose of this study was to assess the psychological impact of instrumental activities of daily living on people with simulated AMD.

**Method:**

Twenty-four normally sighted participants completed 12 household tasks, in a simulated home environment, under a moderate-to-severe AMD simulation. Participants’ psychological state was measured through self-report questionnaires and physiological measurements related to anxiety and stress. Tasks were completed twice, under counterbalanced vision conditions (normal and simulated AMD).

**Results:**

Linear mixed models on vision condition (normal versus simulated AMD) and trial order (trial 1 versus trial 2) revealed a significant large negative effect of the AMD simulation on time to complete tasks, and the anxiety, task engagement and distress self-reports (all *P* < 0.024, all *ω*^2^ > 0.177). There were also significant medium-large effects of trial order on time, task incompletion, task errors, and the anxiety and task engagement self-reports (all *P* < 0.047, all *ω*^2^ > 0.130), whereby the results improved during the second attempt at the tasks. No physiological measures were significant (all *P* > 0.05).

**Conclusions:**

Completing instrumental activities of daily living under an AMD simulation had a negative impact on participants’ self-reported mental state. The observed trial order effects also illuminated how practice with tasks could ease anxiety and stress over time.

## Age-related macular degeneration

The prevalence of mental health problems (e.g. depression, anxiety) is higher in visually impaired people than normally sighted samples.^1,2^ Within the visually impaired, those with age-related macular degeneration (AMD) were shown to have greater declines in psychological well-being than those with other visual impairments.^3^ The pronounced declines may be attributed to AMD's progressive nature and limited treatability.^3,4^

The defining characteristic of AMD is the deterioration of part of the retina (the macula), which leads to gradual loss of central vision.^5^ Central vision loss leaves people with AMD vulnerable to harm as they become disadvantaged at detecting surrounding hazards.^6^ Indeed, Wood et al^7^ found that 74% of a patient group with AMD reported at least one fall or injury over the course of a year, with 30% reporting two or more falls. The stress of potentially hurting oneself can also precipitate fear toward engaging in daily activities, leading to dependence, social isolation and consequently, a reduction in mental health.^8,9^

## Simulating vision loss

A challenge in isolating the psychological effects of vision loss are the comorbidities typically found in AMD. Visual impairments disproportionally affect older adults, and those with vision loss are subject to significantly more physical and mental comorbidities (e.g. stroke, hearing loss, dementia) than those without.^10^ One approach to circumvent these issues is to simulate AMD conditions in normally sighted participants. This strategy enables the disentanglement of specific vison-related effects on behaviours from other age-related declines and comorbid factors.

However, simulation techniques should be employed cautiously. As visual decline, particularly for AMD, typically occurs progressively, patients can learn compensatory strategies (i.e. peripheral retinal locus or eccentric fixation) to adapt to vision loss.^11,12^ Such strategies may not be developed during a short-term simulation study. Moreover, a scotoma moves synchronously with a person's eye, whereas the scotoma in many simulations are stable.^11^ As such, simulations can never fully replicate life with AMD, and may even underestimate the true effect of vision loss.^13^ Nonetheless, findings of simulation research generally mimic behaviours exhibited in studies on patients with AMD.^11^ For example, reduced reading speed resulting from AMD has been identified in both simulation and patient studies.^14,15^ Therefore, an AMD simulation will, at a minimum, provide preliminary insights into the impact of visual impairment on everyday life.

## Impact of activities of daily living

It is well established that visually impaired populations can struggle with activities of daily living, such as walking, recognising faces and driving.^5,16^ Studies assessing how AMD affects instrumental activities of daily living have typically confirmed this through objective measures of task performance (i.e. accuracy, errors, time),^17,18^ but fewer studies have explored the implications of living with AMD from a psychological and physiological perspective. Of the studies that have examined mental health, many findings have been based upon reflective self-reports via longitudinal prospective cohort studies or retrospective case–control studies.^2,19^ Measuring a person's psychological response to everyday tasks has been relatively overlooked in a visual impairment context. However, previous research demonstrates that linking psychological and physiological data is possible,^20–23^ and could therefore be utilised to measure the combined impact of AMD on everyday tasks.

An analysis of the functional status in over 750 patients with AMD identified an increased risk of functional disability for instrumental activities of daily living, like housework.^24,25^ Moreover, a study on daily tasks found that over twice as many patients with AMD reported needing help with housework, or being unable to complete housework even with help, compared with a control group.^25^ There is currently a gap in the literature regarding the psychological state of those with AMD during housework. Despite the familiarity that any person has with such tasks, it should not be presumed that housework may not still be a large source of unacknowledged distress. Visually impaired people have described the additional energy and focus required to undertake tasks with low vision as mentally fatiguing.^26^ Therefore, even when ordinary tasks (i.e. folding towels) have little risk of causing physical harm, they could still negatively contribute to a person's mental health.

Consequently, this study will investigate the short-term psychological and physiological impact of completing instrumental activities of daily living (i.e. housework) in a simulated home environment – with bedrooms, kitchen and living areas. Utilising a real-world environment will be fundamental in generating ecologically valid responses. We hypothesise that participants completing instrumental activities of daily living under an AMD simulation will incur greater psychological and physiological distress than without the simulation.

## Method

### Participants

Participants were recruited by means of advertisements at the University of South Australia. The recruitment criteria included competent English-speaking adults aged 18 years and above, with no history of visual impairments and corrected visual acuity better than 6/18 (Snellen acuity chart). The use of a normally sighted population helped to trial the feasibility of the study before potentially expanding the research into clinical populations. During eligibility screening, potential participants were asked if they had any psychiatric disorders or cognitive impairments. A formal cognitive or medical history assessment was not made at this point. Final eligibility was dependent on participants fitting into a Hexoskin,^27^ a biometric shirt used to collect physiological data. Participants were also subject to COVID-19 requirements (e.g. symptom checks and high-risk health category restrictions) as instituted by the University of South Australia. The authors assert that all procedures contributing to this work comply with the ethical standards of the relevant national and institutional committees on human experimentation and with the Helsinki Declaration of 1975, as revised in 2008. All procedures involving human participants were approved by the University of South Australia's Human Research Ethics Committee (protocol 2028089). Written informed consent was obtained from all participants; and they were offered $30 as honorarium for their participation.

### Apparatus and measures

#### Simulated vision goggles

**Fig. 1:**
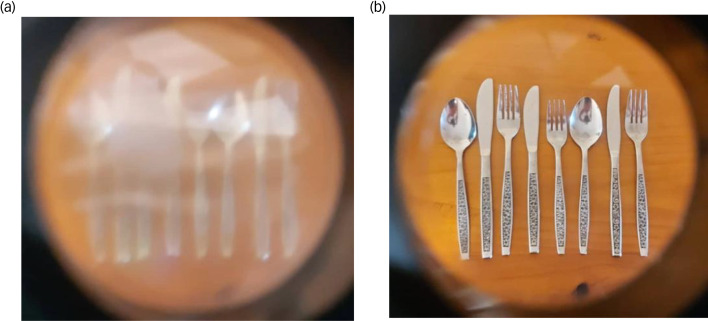
Picture of cutlery taken through the simulated AMD vision and normal vision goggles. (a) Example of simulated AMD vision. (b) Example of normal vision. AMD, age-related macular degeneration.

A moderately severe AMD impairment was simulated via Fork in the Road macular degeneration simulator goggles (see Fig. 1;^28^). Two layers of 20 mm diameter circular Bangerter occlusion foils of 0.1 LogUnit (resulting in 6/60 vision) were then added to create a 10° central scotoma monocularly in both eyes. These were placed in the inner central region of each lens. A neuro-ophthalmologist calibrated the goggles to verify that the visual effect reliably reflected 6/60 best corrected visual acuity in either eye. The scotoma was verified with Zeiss Humphry 24-2 automated visual field analyser (Carl Zeiss Meditec Inc., Jena, Germany).

#### Normal vision goggles

The same Fork in the Road macular degeneration simulator goggles^28^ were utilised. Except, the elements used for creating the AMD visual effect were removed from the goggles, leaving only a clear lens. Therefore, the normal vision goggles were not intended to alter participant's vision at all. The use of the goggles in both conditions ensured equity in wearing the goggle frames (i.e. weight, comfort, shape, same level of restricted peripheral vision).

#### Activities of daily living

**Fig. 2:**
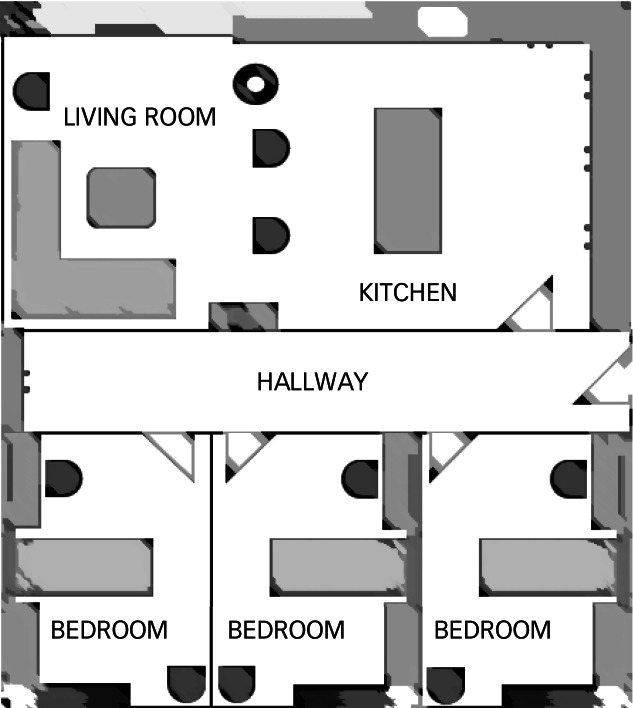
Sleep and Chronobiology Laboratory. The Sleep and Chronobiology Laboratory is within the MC building at Magill Campus, University of South Australia. From left to right/up to down: the rooms used in the study included the living room and kitchen (35 m^2^), hallway (18.9 m^2^) and bedrooms (11.4 m^2^ each).

The study was conducted at the University of South Australia's Sleep and Chronobiology Laboratory (see Fig. 2), which emulates a house environment. This study used three of the bedrooms, the hallway, living room and kitchen, to allow participants to engage in instrumental activities of daily living.

Twelve tasks that required mobility around the laboratory and visual discrimination of objects (e.g. colour and texture) were developed (see Table 1). Each set of tasks was completed twice, using the normal and simulated AMD goggles.

**Table 1:** Instructions for the 12 instrumental activities of daily living

Task instructions
Locate the unmade bed and make the bed properly. Fold the three black towels on the kitchen table, then place a towel on the end of each bed. Pick up three grey pillows in the hallway and return to the living room couch. Return the magazines in the living room to the stand on the wall. Pick up the three books in each bedroom and stack them on the bedside tables. Collect the three fluffy toys from the hallway and place on the game stack in the living room. Sort the cutlery (forks, knives and spoons) on the kitchen bench into individual piles. Locate the blue coloured cups in each bedroom and place in the kitchen sink. Collect the full rubbish bin from the hallway and place next to the large rubbish bin in the kitchen. Arrange the animal picture cards in the living room into a neat pile. Pick up the six plastic blocks on the kitchen floor and place on the kitchen table. Collect the DVD from each bedroom and place next to the living room TV.

**Fig. 3:**
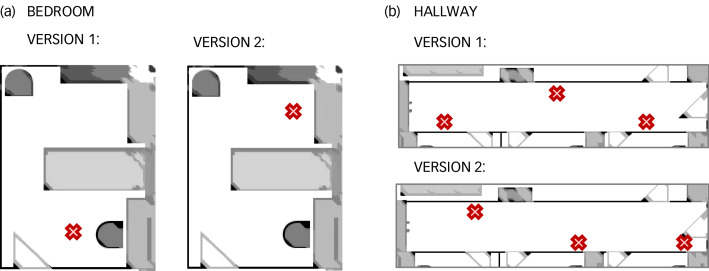
Alterantive material locations. (a) Example of the alternative locations of the DVDs in each bedroom. (b) Example of the alternative locations of the pillows in the hallway.

To reduce the impact of practice effects between the normal and simulated AMD conditions, there were two versions of the location of the materials and two versions of the instructions. As illustrated in Fig. 3, the location of materials within the laboratory could differ by finding an item under a bedroom desk versus by the bedroom door. This forced participants to visually search for items during the second task set instead of relying on their previous exposure. Likewise, the task instructions could differ by colour or material item (see Supplementary Appendix 1 available at https://doi.org/10.1192/bjo.2022.558 for alternative instructions). For example, ‘Fold the three black towels … ’ versus ‘Fold the three pink towels … ’; or ‘Arrange the animal picture cards … ’ versus ‘Arrange the Uno number cards … ’. Once again, this required participants to apply visual discrimination during the task sets. Although the order of vision condition (i.e. normal vision completed first or second) was counterbalanced between participants, the material locations and task instructions were randomised to minimise learning effects.

#### Performance measures

Two task performance measures were interpreted from the Hexoskin. This included the time taken from the start of the first task until the end of the last task, and the number of steps. An increase in either measure was inferred as reduced task performance, because it suggested participants put greater effort into completing the task (e.g. more time spent searching for an item because of disrupted vision). The researcher also noted two observational measures during the testing session: task incompletion (whether the task was completed) and task errors (how many errors were made during the task). Task errors included any deviation from the specific instruction (e.g. missed materials, collecting incorrect coloured materials, moving to incorrect locations). Increased difficulty completing the task would be indicated by increased task incompletion and more task errors.

#### Psychological measures

The State–Trait Anxiety Inventory (STAI) and the Short Stress State Questionnaire (SSSQ) are two self-report questionnaires administered to capture anxiety and stress, respectively. In terms of the STAI, only the State form was used; a 20-item scale examining the presence and severity of current symptoms of anxiety.^29^ The higher the score, the greater the presence of anxiety. The STAI-State has good internal consistency: α = 0.86–0.95.^29^

The SSSQ is a 24-item measure comprising three subscales: worry (cognitive interference and self-regulation), task engagement (motivation and task focus) and distress (negative affect and mood). Higher worry and distress scores, and lower task engagement scores suggest the presence of stress. The SSSQ has good internal consistency in each domain: α = 0.84, α = 0.81 and α = 0.87, respectively.^30^

#### Physiological measures

Recordings were collected with a Hexoskin biometric shirt.^27^ There were eight Hexoskin shirts available representing different sizes (one S and XXL, as well as two each for M, L and XL). The shirt comprises electrocardiogram, respiratory inductive plethysmography and accelerometer sensors, to compute cardiac (sampling rate: 256 Hz), respiratory (128 Hz) and activity (64 Hz) data, respectively. The sensors were embedded within the shirt, across the thorax and navel regions. This recording method was unobtrusive and comfortably worn by participants under their clothing. The data was saved to a recording device fitted within the shirt during testing. The device was removed post-testing, and data was uploaded to the Hexoskin Online Dashboard. Use of the Hexoskin in this manner has been validated by similar studies.^20,31^

#### Debriefing

Participants were asked simple follow-up questions upon completion of the experiment to subjectively ascertain which vision condition (normal, simulated AMD or neither) was more difficult and triggered greater anxiety or stress.

### Procedure

Participants acclimated to the vision goggles before the main task by completing two cognitive tasks under each condition (normal and simulated AMD; results reported in Macnamara et al^32^). This adaptation period lasted approximately 30 mins.

**Fig. 4:**
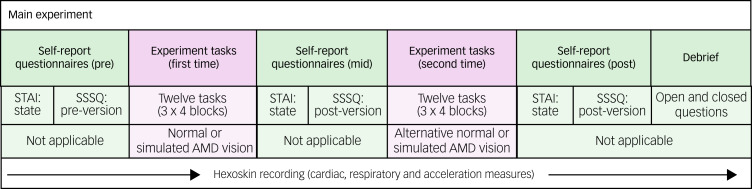
Study procedure. AMD, age-related macular degeneration; SSSQ, Short Stress State Questionnaire; STAI, State–Trait Anxiety Inventory.

As outlined in Fig. 4, the subsequent main experiment was split into two task blocks, with the psychological questionnaires administered before, during and after the instrumental activities of daily living tasks. During the first block of tasks, participants wore either the normal or simulated AMD goggles to complete the tasks. During the subsequent second block, the tasks were repeated under the alternative vision condition. Participants could wear prescription glasses beneath the goggles, if required.

Within each experimental task block, participants completed the 12 tasks (see Table 1). These were split into a further four sub-blocks (three tasks per block) for ease of remembering the required tasks. At the start of each sub-block, the researcher read the instructions for three tasks aloud (twice), and participants were required to accurately repeat the instructions back to demonstrate comprehension. Instructions were not offered again unless requested. Instead, prompts were provided from one task to the next (e.g. ‘*Return cups to sink’*). Once the three tasks were completed, the participants were provided with the three task instructions for the next sub-block. This continued until all four sub-blocks (and consequently all 12 tasks) were completed.

The entire study took place during a single session and lasted for approximately 90 min. Participants wore the Hexoskin for the duration of the session and were debriefed at the end.

### Data processing

Data was processed with RStudio (version 1.3.1093 for Windows; RStudio, Boston, USA, https://www.rstudio.com/products/rstudio/download/). The raw physiological data was downloaded from the Hexoskin Dashboard, including time taken to complete the tasks, steps, the R–R interval, heart rate and expiration timing.

Signal quality assessments for the R–R interval were used to exclude participants from analyses when >20% of the data was noisy or unreliable, according to the Hexoskin quality measures included in the software package.^33^ The R–R interval was further pre-processed with the R Heart Rate Variability (RHRV) package in R.^34^ Artifacts were removed via the RHRV filter function, whereby outlier parameters were set to 1.25 times the interquartile range. Any remaining outliers were manually removed with the RHRV edit function. The heart rate was then interpolated and heart rate variability time domain (standard deviation of NN intervals (SDNN) and the root mean square of successive differences (rMSSD)) and frequency domain analyses (low-frequency (0.05–0.15 Hz) and high-frequency (0.15–0.4 Hz) relative measures) were created. Lower SDNN and rMSSD are indicative of a greater stress response.^20^ Moreover, stress is recognised by an increase in low-frequency and decrease in high-frequency activity.^22^

The expiration data were processed into an expression of respiratory rate, calculated as the average number of seconds between breaths. A reduction in the number of seconds between breaths was interpreted as increased anxiety, because the reduction in seconds indicates an increase in respiratory rate.^21^

The STAI-State was scored by reverse-coding selected items (as indicated by the scale guidelines), then summing the scores for all of the items.^29^ The SSSQ was scored according to the method presented in Helton and Näswall,^35^ in which the mean score per subscale was calculated. Henceforth, the questionnaire scores will be referred to as the anxiety (STAI-State), worry, task engagement and distress (SSSQ) scales.

### Statistical analysis

All statistical analyses were conducted in Jamovi (version 1.6 for Windows; The jamovi project, Sydney, Australia, https://www.jamovi.org/download.html). Significance was set at *P <* 0.05 for all tests. Outliers – more than three standard deviations above or below the mean – were excluded from analysis. Separate linear mixed models were conducted to analyse the following dependent variables: performance measures of steps, time, task completion and task errors; psychological measures of anxiety, worry, task engagement and distress; and physiological measures of heart rate, SDNN, rMSSD, low frequency, high frequency and respiratory rate. In each model, the participant identification number was set as a random intercept. Vision condition (normal versus simulated AMD) and trial order (trial 1 versus trial 2) were entered as predictor variables. For the psychological data (i.e. anxiety, worry, task engagement and distress), additional baseline scores for each of the scales were added as covariates. Preliminary models were conducted controlling for age and gender, but since these variables did not change the results, age and gender were not included as covariates.

When appropriate, Bonferroni-corrected *post hoc* comparisons were conducted. Effect sizes (*ω^2^*) were calculated with the effect size online package,^36^ and interpreted as very small (<0.01), small (0.01–0.06), medium (0.06–0.14) and large (>0.14), according to Field.^37^

## Results

Forty-three people indicated their interest in the study. Two of these people did not complete the screening process, and seven people did not fulfil all of the inclusion criteria: one was excluded for history of psychiatric disorders, three for COVID-19 restrictions (e.g. high-risk health categories) and three for Hexoskin sizes. Of the remaining 34 people, ten were unable to attend testing because of scheduling issues and developing COVID-19 symptoms the day before testing. A total of 24 participants (19 women, five men; age range 18–60 years, mean 27.1 years, s.d. 9.7 years), completed this study. The linear mixed-model results for the performance, psychological and physiological measures are presented in Table 2. The corresponding marginal means, s.e. and 95% confidence intervals for the vision condition and trial order variables are reported in Supplementary Appendix 2.

**Table 2:** Linear mixed-model results of the effect of vision condition and trial order

Dependent variable	Predictor^a^	Estimate	s.e.	*t*-value	*P*-value	Effect size (*ω^2^*)
Performance						
Time	Vision	**108**.**01**	**17**.**67**	**6**.**11**	**<0**.**001**	**0**.**609**
	Order	**−131**.**51**	**17**.**67**	**−7**.**44**	**<0**.**001**	**0**.**700**
	Interaction	0.977	59.10	0.017	0.987	<0.001
Steps	Vision	−0.667	10.18	−0.066	0.948	<0.001
	Order	0.167	10.18	0.016	0.987	<0.001
	Interaction	7.50	61.05	0.123	0.903	<0.001
Task incompletion	Vision	0.125	0.260	0.482	0.635	<0.001
	Order	**−1**.**22**	**0**.**260**	**−4**.**66**	**<0**.**001**	**0**.**463**
	Interaction	−0.417	0.541	−0.771	0.449	<0.001
Task errors	Vision	0.208	0.263	0.792	0.437	<0.001
	Order	**−1**.**63**	**0**.**263**	**−6**.**17**	**<0**.**001**	**0**.**607**
	Interaction	−0.583	1.39	−0.420	0.679	<0.001
Psychological						
Anxiety	Vision	**4**.**42**	**1**.**82**	**2**.**44**	**0**.**024**	**0**.**177**
	Order	**−4**.**24**	**1**.**82**	**−2**.**34**	**0**.**029**	**0**.**162**
	Baseline	**0**.**562**	**0**.**189**	**2**.**97**	**0**.**007**	**0**.**263**
	Interaction	4.21	6.55	0.643	0.528	<0.001
Worry	Vision	0.082	0.078	1.05	0.307	0.004
	Order	−0.104	0.078	−1.34	0.195	0.033
	Baseline	**0**.**830**	**0**.**095**	**8**.**73**	**<0**.**001**	**0**.**774**
	Interaction	−0.512	0.312	−1.64	0.117	0.071
Task engagement	Vision	**−0**.**175**	**0**.**051**	**−3**.**45**	**0**.**002**	**0**.**322**
	Order	**0**.**107**	**0**.**051**	**2**.**11**	**0**.**047**	**0**.**130**
	Baseline	**0**.**940**	**0**.**152**	**6**.**19**	**<0**.**001**	**0**.**629**
	Interaction	−0.128	0.334	−0.384	**0**.705	<0.001
Distress	Vision	**0**.**207**	**0**.**074**	**2**.**79**	**0**.**015**	**0**.**304**
	Order	−0.116	0.074	−1.56	**0**.141	0.085
	Baseline	0.092	0.187	0.492	0.630	<0.001
	Interaction	−0.180	0.190	−0.947	0.361	<0.001
Physiological						
Heart rate	Vision	−0.229	0.567	−0.405	0.690	<0.001
	Order	0.336	0.567	0.593	0.559	<0.001
	Interaction	−5.03	9.70	−0.518	0.609	<0.001
SDNN	Vision	−1.30	2.49	−0.524	0.607	<0.001
	Order	2.26	2.49	0.909	0.377	<0.001
	Interaction	29.34	21.90	1.34	0.195	0.034
rMSSD	Vision	−0.831	2.21	−0.376	0.712	<0.001
	Order	1.32	2.21	0.596	0.560	<0.001
	Interaction	13.51	12.15	1.11	0.279	0.011
Low-frequency analysis	Vision	820.83	2358.88	0.348	0.734	<0.001
	Order	−352.78	2358.88	−0.150	0.884	<0.001
	Interaction	5167.70	8077.96	0.640	0.532	<0.001
High-frequency analysis	Vision	140.78	220.65	0.638	0.535	<0.001
	Order	−167.86	220.65	−0.761	0.462	<0.001
	Interaction	642.16	832.36	0.772	0.452	<0.001
Respiratory rate	Vision	0.017	0.070	0.240	0.813	<0.001
	Order	−0.063	0.070	−0.898	0.381	<0.001
	Interaction	−0.795	0.380	−2.09	0.050	0.137

Significant predictors (*P* < 0.05) are in bold.

a. All interaction predictors reflect a vision condition × trial order interaction.

### Performance data

**Fig. 5:**
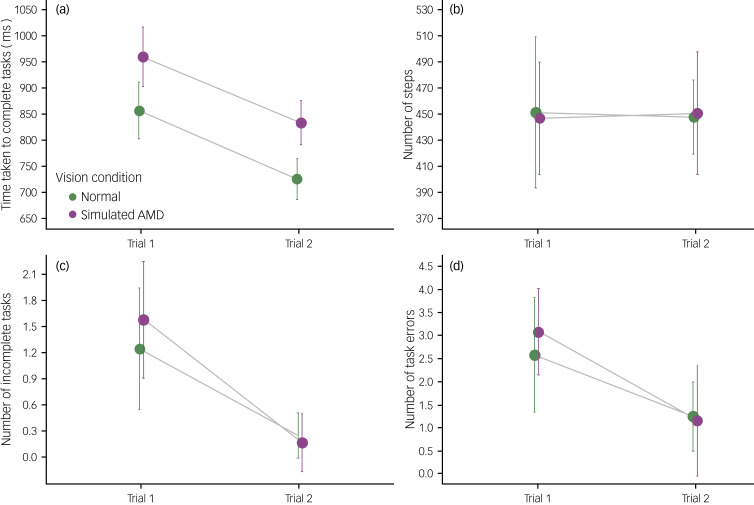
Vision condition (normal versus simulated AMD) and trial order (trial 1 versus trial 2) effects for the task performance measures. Results are for (a) time, (b) steps, (c) task incompletion and (d) task errors. AMD, age-related macular degeneration.

There was a significant main effect of vision condition for time (*F*(1, 21.31) = 37.35, *P* < 0.001, *ω^2^* = 0.609). As illustrated in Fig. 5(a), participants were slower to complete the tasks during the simulated AMD condition (mean 900.93 s, s.e. 17.36) compared with the normal vision condition (mean 792.92 s, s.e. 17.07). There was also a significant main effect of trial order (*F*(1, 21.31) = 55.37, *P* < 0.001, *ω^2^* = 0.7), with participants completing the tasks slower during their first trial (mean 912.68 s, s.e. 17.36) compared with their second trial (mean 781.17 s, s.e. 17.07). There was no significant interaction between vision condition and trial order (*P* = 0.987).

There were no significant main effects or interaction for the number of steps taken to complete the tasks (all *P* > 0.903; see Fig. 5(b)).

Regarding task incompletion, results of a linear mixed model revealed a significant main effect of trial order (*F*(1, 22) = 21.67, *P* < 0.001, *ω^2^* = 0.463). Tasks were more often incomplete during the first trial (mean 1.42, s.e. 0.187) compared with the second trial (mean 0.208, s.e. 0.187; see Fig. 5(c)). The was no significant main effect of vision condition (*P* = 0.635), nor interaction between vision condition and trial order (*P* = 0.449).

There was a significant main effect of trial order for task errors (*F*(1, 22) = 38.11, *P* < 0.001, *ω^2^* = 0.607). Figure 5(d) shows that task errors occurred more during the first trial (mean 2.83, s.e. 0.372) compared with the second trial (mean 1.21, s.e. 0.372). There was no significant main effect of vision condition (*P* = 0.437), nor interaction between vision condition and trial order (*P* = 0.679).

### Psychological data

**Fig. 6:**
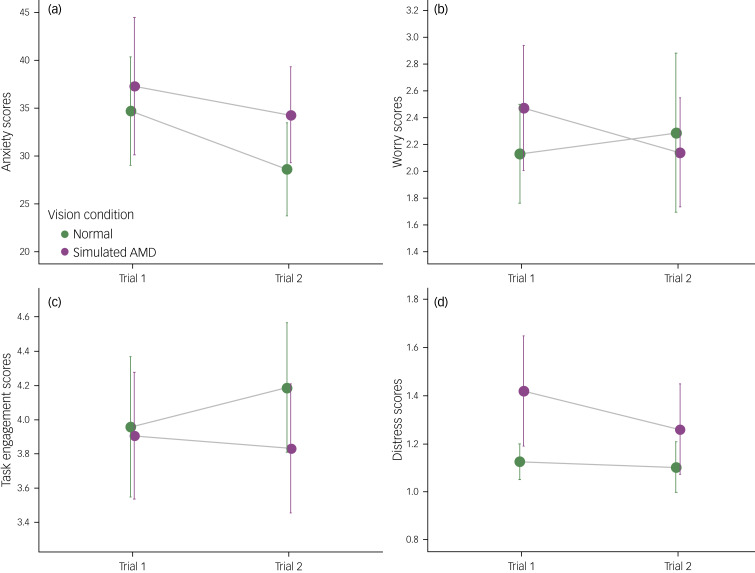
Vision condition (normal versus simulated AMD) and trial order (trial 1 versus trial 2) effects for the psychological measures. Results models are for (a) anxiety, (b) worry, (c) task engagement and (d) distress. Higher scores on (a), (b) and (d), and lower scores on (c), suggest a ‘negative’ response. AMD, age-related macular degeneration.

Results of a linear mixed model found a significant main effect of vision condition for anxiety (*F*(1, 21) = 5.93, *P* = 0.024, *ω^2^* = 0.177). The anxiety scores were higher after the simulated AMD condition (mean 35.91, s.e. 1.87) than after the normal vision condition (mean 31.49, s.e. 1.87; see Fig 6(a)). There was also a significant main effect of trial order (*F*(1, 21) = 5.46, *P* = 0.029, *ω^2^* = 0.162), whereby anxiety scores were higher during the first trial (mean 35.82, s.e. 1.87) compared with the second trial (mean 31.58, s.e. 1.87). Baseline scores significantly predicted subsequent anxiety scores (*F*(1, 20) = 8.85, *P* = 0.007, *ω^2^* = 0.263). There was no significant interaction between vision condition and trial order (*P* = 0.528).

In terms of worry, baseline scores significantly predicated subsequent worry scores (*F*(1, 20) = 76.17, *P* < 0.001, *ω^2^* = 0.774). There were no significant main effects or interaction (all *P* > 0.117; see Fig. 6(b)).

There was a significant main effect of vision condition for task engagement (*F*(1, 21) = 11.93, *P* = 0.002, *ω^2^* = 0.322). As illustrated in Figure 6(c), task engagement scores after the simulated AMD condition were lower (mean 3.87, s.e. 0.087) than after the normal vision condition (mean 4.05, s.e. 0.087). There was also a significant main effect of trial order (*F*(1, 21) = 4.43, *P* = 0.047, *ω^2^* = 0.130). Scores for task engagement were higher during the second trial (mean 4.01, s.e. 0.087) compared with the first trial (mean 3.91, s.e. 0.087). Baseline scores significantly predicted subsequent task engagement scores (*F*(1, 20) = 38.34, *P* < 0.001, *ω^2^* = 0.629). There was no significant interaction between vision condition and trial order (*P* = 0.705).

Regarding distress, a linear mixed model found a significant main effect of vision condition (*F*(1, 13.53) = 7.79, *P* = 0.015, *ω^2^* = 0.304). Figure 6(d) displays that distress scores were higher after the simulated AMD condition (mean 1.32, s.e. 0.060) than after the normal vision condition (mean 1.11, s.e. 0.060). There was no significant main effects of trial order (*P* = 0.141), nor interaction between vision condition and trial order (*P* = 0.361). Baseline scores did not significantly predict subsequent distress scores (*P* = 0.630).

### Physiological data

The linear mixed models yielded no significant main effects nor interactions for the heart rate, SDNN, rMSSD, low frequency, high frequency and respiratory rate data (all *P* > 0.05; see Supplementary Appendix 3 for figure).

### Debriefing

Based on feedback following the tasks, approximately 92% of participants felt that it was more difficult to finish the tasks during the simulated AMD condition (see Table 3). Also, approximately 83% of participants experienced greater anxiety or stress during the simulated AMD condition.

**Table 3:** Participant's responses to follow-up questions

Questions	Response count		
	Normal vision	Simulated AMD vision	Neither
Under which condition was it more difficult to finish the tasks?	0	22	2
Under which condition did you experience more anxiety or stress?	1	20	3

AMD, age-related macular degeneration.

## Discussion

In this study, we investigated the psychological impact of instrumental activities of daily living (i.e. housework) on people with simulated AMD. There were a series of main effects for task performance and the psychological self-reports, but no physiological measures reached significance. Critically, AMD simulation does have a significant negative impact on symptoms associated with a person's mental health during everyday tasks.

Regarding task performance, participants took significantly longer to complete the activities in the simulated AMD condition, mimicking findings from other daily tasks and wayfinding simulation studies.^17,38^ Despite this outcome, participants’ competency (i.e. completion rate and errors) in completing the tasks did not change by vision condition, demonstrating that people with moderately severe AMD can still successfully complete tasks, but with reduced functioning speed. The outcome coincides with the participants’ self-reported engagement with the tasks. Task engagement scores were significantly lower during the simulated AMD condition, suggesting that loss of vision may affect a person's motivation and confidence in their abilities to complete the activities. Therefore, the participants may have undertaken the tasks at a slower pace to ensure that they were being done correctly. Our observations expand the findings of other visual impairment research on speed and accuracy. For example, reading performance studies have previously found that participants’ reading speed decreases as the degree of visual impairment (e.g. scotoma size) increases.^39^ Essentially, as vision declines, people forgo time so as to complete an activity correctly. Speed–accuracy trade-offs have already been posited to explain slower visual processing speeds, particularly within ageing populations.^40^

Completing tasks under vision loss conditions was also found to have a large negative impact on the mental state of participants. As a direct result of the AMD simulation, there were significantly worse anxiety, task engagement and distress scores. Of note, the distress scale is a specific measure of negative affect and mood, which only underscores the degree to which everyday tasks can influence the mental state of a visually impaired person. Participants’ final feedback was also consistent with the psychological scores, with 83% of participants responding that they experienced more anxiety and stress during the simulated AMD condition. As vision loss was simulated in a younger population without age-related comorbidities (i.e. cognitive decline, hearing loss), we believe that these findings can reasonably be attributed to the effects of vision loss.

The results also revealed that repetition of the tasks greatly influenced the participants behaviour. Performance on the tasks significantly improved (i.e. reduced time, more tasks completed, less task errors) during the participants’ second attempts at completing them. This is not unusual given that practice effects are present in repeated measures designed experiments.^41^ But, interestingly, there were medium-large effects of trial order for self-reported anxiety and task engagement as well. Although this could also be a simple effect of practice, it may also reaffirm vision-related rehabilitation strategies. Patients with AMD may be encouraged during rehabilitation by the knowledge that repeatedly undertaking a task may lessen their anxiety and build confidence in their abilities.

A strength of this study is that it is one of the first, to our knowledge, that obtains physiological and psychological data to investigate how AMD affects aspects of mental health during instrumental activities of daily living. However, the observed changes in physiological data did not reach significance. The disparity between this data and the psychological scores may be attributed to the requirements of the study tasks. Completing daily tasks (e.g. folding towels) may be considerably less alarming than the activities in previous studies that showed a physiological stress response. For example, participants in a ‘stressful’ virtual reality simulation were instructed to ride an open elevator to the top of a building and then step off the edge.^23^ Compared with the control condition (riding a virtual closed elevator to the third floor), physiological recordings indicated a greater stress response (e.g. increased pulse rate, skin conductance, salivary cortisol and changes in heart rate variability^23^). Therefore, the non-significant change in our study could simply be because household tasks do not trigger a measurable difference in physiological responses.

Although the physiological measures did not reflect the increased anxiety and stress the participants self-reported in the AMD condition, our findings should not be used to dismiss the use of physiological measures in visual impairment research. Research into activities of daily living with different population groups have already been incorporating physiological measures (e.g. limb amputees or patients with lung disease).^42,43^ Furthermore, the use of physiological and objective wearable measures, such as the Hexoskin, can broaden the scope of research from studies investigating specific triggers (i.e. reading, housework) to unconstrained field work.

As discussed, limitations of the current study include the nature of the tasks. Although we were specifically interested in familiar instrumental activities of daily living, like housework, we recognise that physiological differences may not be discernible unless repeated with tasks that have been identified as concerning for people with AMD (e.g. stair climbing).^44^ Furthermore, although the use of younger, corrected to normally sighted people under simulation was intended to reduce the influence of comorbidities, it still needs to be acknowledged that this is not the demographic (i.e. typically those aged over 45 years) most afflicted by AMD.^45^ Consequently, the sample related constraints of this study, including gender imbalance, small sample size, participant age group and visual status, may have hindered the generalisability of this study and should be addressed in future research.

In terms of the simulation, the adaption period for the AMD simulation goggles was approximately 30 mins, which does not equate to the long-term (i.e. months, years) oculomotor adaptations that may occur if patients develop a peripheral retinal locus to compensate for central vision loss.^11,12^ Therefore, this study could have overestimated the psychological effects of AMD (e.g. increased anxiety and distress), since the normally sighted participants had far less time to adjust their behaviour. This is also reflected by the effects of trial order, which imply that the psychological effects may improve with practice. Opposingly, it is also possible that the study underestimated the psychological effects of AMD if participants circumvented the simulated scotoma by looking elsewhere through the simulation goggles.^11^ In such as case, the normally sighted participants may not have experienced the same level of visual disability that patients with AMD have.

In summary, this is the first study, to our knowledge, where a person's mental health during instrumental activities of daily living were measured psychologically and physiologically in response to simulated AMD. There was a significant large effect on self-reported anxiety, stress and completion time as a direct result of simulated vision loss. Since completing household tasks occurs daily over a long-term period, it is conceivable that instrumental activities of daily living do contribute negatively to the mental health of visually impaired people. However, clinical research on patients with AMD in the future could help to further elucidate this relationship.

## Data Availability

The data that support the findings of this study are available from the corresponding author, A.M., upon reasonable request.

## Supplementary material

For supplementary material accompanying this paper visit http://doi.org/10.1192/bjo.2022.558. click here to view supplementary material
