# Supplementary material for: The psychological impact of instrumental activities of daily living on people with simulated age-related macular degeneration
Source: BJPsych Open. 2022 Aug 8;8(5):e152. doi: 10.1192/bjo.2022.558 (PMC9380024; doi:10.1192/bjo.2022.558)
Supplement: Supplementary file 1 [file S2056472422005580sup001.docx]

**Appendices**

**Appendix A:**

Alternative full task instructions

1. Locate the unmade bed and make the bed properly.

2. Fold the three pink towels on the kitchen table, then place a towel on the end of each bed.

3. Pick up three blue pillows in the hallway and return to the living room couch.

4. Return the magazines in the living room to the stand on the wall.

5. Pick up the three books in each bedroom and stack them on the bedside tables.

6. Collect the three fluffy toys from the hallway and place on the game stack in the living room.

7. Sort the cutlery (forks, knives, and spoons) on the kitchen bench into individual piles.

8. Locate the clear coloured cups in each bedroom and place in the kitchen sink.

9. Collect the full rubbish bin from the hallway and place next to the large rubbish bin in the kitchen.

10. Arrange the uno number cards in the living room into a neat pile.

11. Pick up the six wooden blocks on the kitchen floor and place on the kitchen table.

12. Collect the DVD from each bedroom and place next to the living room TV.

**Appendix B**

Marginal means, standard errors, and confidence intervals for vision condition.

| **Measure** | **Vision Condition** | **Mean** | **SE** | **95% Confidence Interval** | |
| --- | --- | --- | --- | --- | --- |
|  |  |  |  | **Lower** | **Upper** |
| **Performance** |  |  |  |  |  |
| Time | Normal | 792.92 | 17.07 | 758.27 | 827.57 |
|  | Simulated AMD | 900.93 | 17.36 | 865.71 | 936.15 |
| Steps | Normal | 450.29 | 16.09 | 417.27 | 483.31 |
|  | Simulated AMD | 449.62 | 16.09 | 416.60 | 482.65 |
| Task incompletion | Normal | 0.750 | 0.187 | 0.372 | 1.13 |
|  | Simulated AMD | 0.875 | 0.187 | 0.497 | 1.25 |
| Task errors | Normal | 1.92 | 0.372 | 1.16 | 2.68 |
|  | Simulated AMD | 2.13 | 0.372 | 1.36 | 2.89 |
| **Psychological** |  |  |  |  |  |
| Anxiety | Normal | 31.49 | 1.87 | 27.67 | 35.30 |
|  | Simulated AMD | 35.91 | 1.87 | 32.10 | 39.72 |
| Worry | Normal | 2.20 | 0.087 | 2.02 | 2.38 |
|  | Simulated AMD | 2.28 | 0.087 | 2.10 | 2.46 |
| Task engagement | Normal | 4.05 | 0.087 | 3.87 | 4.22 |
|  | Simulated AMD | 3.87 | 0.087 | 3.69 | 4.05 |
| Distress | Normal | 1.11 | 0.060 | 0.987 | 1.23 |
|  | Simulated AMD | 1.32 | 0.060 | 1.19 | 1.44 |
| **Physiological** |  |  |  |  |  |
| HR | Normal | 96.46 | 2.44 | 91.41 | 101.52 |
|  | Simulated AMD | 96.24 | 2.44 | 91.18 | 101.29 |
| SDNN | Normal | 84.46 | 5.60 | 72.86 | 96.06 |
|  | Simulated AMD | 83.16 | 5.63 | 71.52 | 94.80 |
| rMSSD | Normal | 32.15 | 3.21 | 25.53 | 38.77 |
|  | Simulated AMD | 31.32 | 3.25 | 24.64 | 38.00 |
| LF analysis | Normal | 7718.53 | 2357.26 | 2891.93 | 12545.14 |
|  | Simulated AMD | 8539.36 | 2319.95 | 3781.37 | 13297.35 |
| HF analysis | Normal | 663.61 | 237.20 | 176.38 | 1150.84 |
|  | Simulated AMD | 804.39 | 233.85 | 323.10 | 1285.68 |
| Respiratory rate | Normal | 3.23 | 0.102 | 3.02 | 3.44 |
|  | Simulated AMD | 3.25 | 0.101 | 3.04 | 3.45 |

Marginal means, standard errors, and confidence intervals for trial order.

| **Measure** | **Trial Order** | **Mean** | **SE** | **95% Confidence Interval** | |
| --- | --- | --- | --- | --- | --- |
|  |  |  |  | **Lower** | **Upper** |
| **Performance** |  |  |  |  |  |
| Time | Trial 1 | 912.68 | 17.36 | 877.46 | 947.90 |
|  | Trial 2 | 781.17 | 17.07 | 746.52 | 815.82 |
| Steps | Trial 1 | 449.88 | 16.09 | 416.85 | 482.90 |
|  | Trial 2 | 450.04 | 16.09 | 417.02 | 483.06 |
| Task incompletion | Trial 1 | 1.42 | 0.187 | 1.04 | 1.79 |
|  | Trial 2 | 0.208 | 0.187 | -0.169 | 0.59 |
| Task errors | Trial 1 | 2.83 | 0.372 | 2.07 | 3.59 |
|  | Trial 2 | 1.21 | 0.372 | 0.45 | 1.97 |
| **Psychological** |  |  |  |  |  |
| Anxiety | Trial 1 | 35.82 | 1.87 | 32.01 | 39.63 |
|  | Trial 2 | 31.58 | 1.87 | 27.77 | 35.39 |
| Worry | Trial 1 | 2.29 | 0.087 | 2.11 | 2.47 |
|  | Trial 2 | 2.19 | 0.087 | 2.01 | 2.37 |
| Task engagement | Trial 1 | 3.91 | 0.087 | 3.73 | 4.08 |
|  | Trial 2 | 4.01 | 0.087 | 3.83 | 4.19 |
| Distress | Trial 1 | 1.27 | 0.060 | 1.15 | 1.39 |
|  | Trial 2 | 1.15 | 0.060 | 1.03 | 1.28 |
| **Physiological** |  |  |  |  |  |
| HR | Trial 1 | 96.18 | 2.44 | 91.13 | 101.24 |
|  | Trial 2 | 96.52 | 2.44 | 91.46 | 101.58 |
| SDNN | Trial 1 | 82.68 | 5.58 | 71.12 | 94.24 |
|  | Trial 2 | 84.94 | 5.65 | 73.26 | 96.62 |
| rMSSD | Trial 1 | 31.08 | 3.18 | 24.51 | 37.65 |
|  | Trial 2 | 32.40 | 3.28 | 25.67 | 39.12 |
| LF analysis | Trial 1 | 8305.34 | 2272.83 | 3633.20 | 12977.47 |
|  | Trial 2 | 7952.56 | 2402.72 | 3040.96 | 12864.16 |
| HF analysis | Trial 1 | 817.93 | 229.61 | 344.08 | 1291.79 |
|  | Trial 2 | 650.07 | 241.30 | 155.40 | 1144.74 |
| Respiratory rate | Trial 1 | 3.27 | 0.102 | 3.06 | 3.48 |
|  | Trial 2 | 3.21 | 0.101 | 3.00 | 3.41 |

**Appendix C**

| *Vision Condition (Normal Vs Simulated AMD) Effects for the Physiological Measures* |
| --- |
| 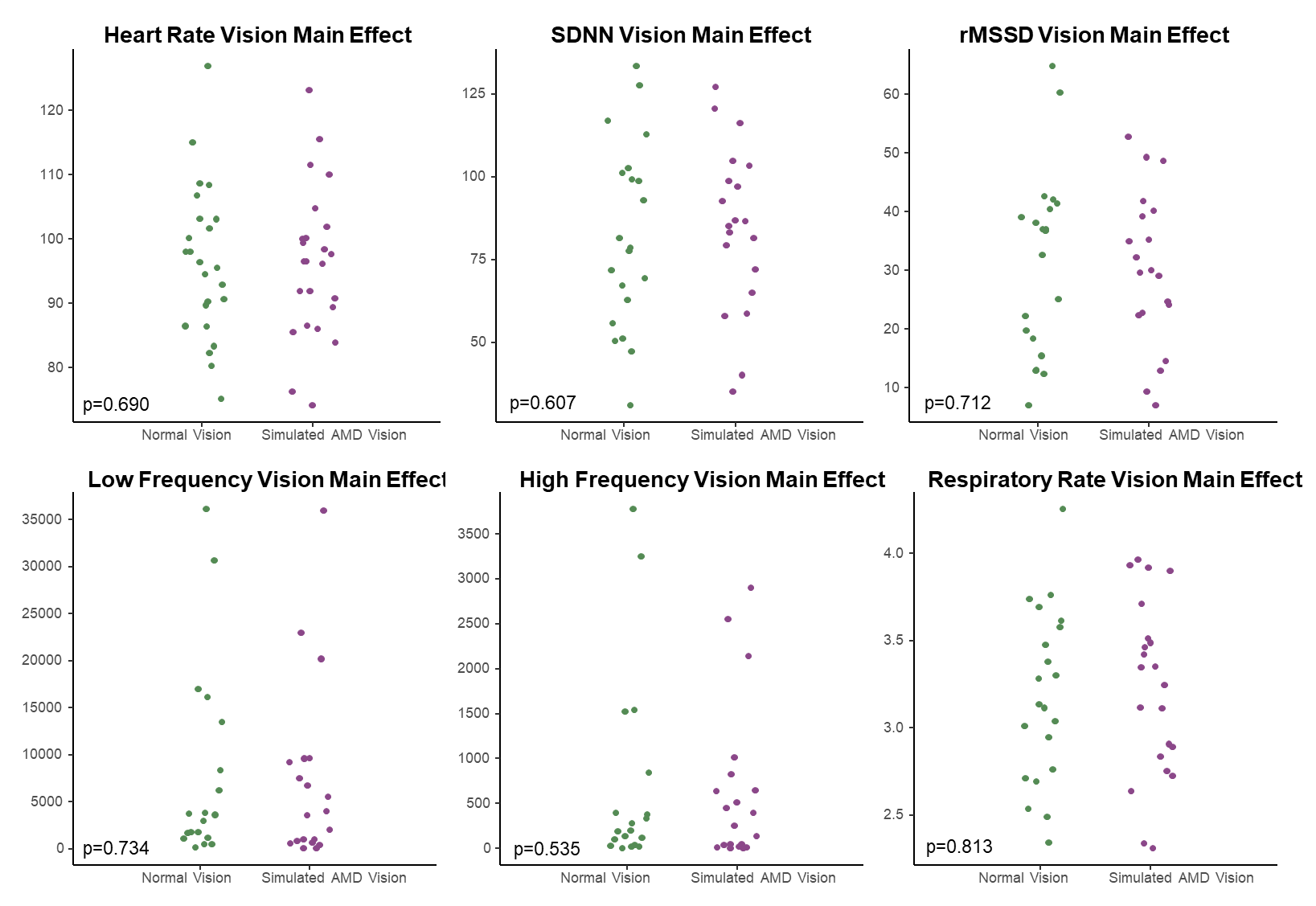 |
|  |

Alt Text. Six scatterplot graphs layed out in two rows of three. Each scatterplot presents data points positioned vertically (in accordance with the y-axis measurement), split by vision condition.
